# Low disabled-2 expression promotes tumor progression and determines poor survival and high recurrence of esophageal squamous cell carcinoma

**DOI:** 10.18632/oncotarget.8460

**Published:** 2016-03-29

**Authors:** Wen-Lun Wang, Wei-Lun Chang, Hsiao-Bai Yang, Yu-Chi Wang, I-Wei Chang, Ching-Tai Lee, Chi-Yang Chang, Jaw-Town Lin, Bor-Shyang Sheu

**Affiliations:** ^1^ Institute of Clinical Medicine, National Cheng Kung University Medical Center, Tainan, Taiwan; ^2^ Department of Internal Medicine, National Cheng Kung University Hospital, Tainan, Taiwan; ^3^ Department of Pathology, National Cheng Kung University Hospital, Tainan, Taiwan; ^4^ Department of Pathology, Ton-Yen General Hospital, Hsin-Chu, Taiwan; ^5^ Department of Biological Science & Technology, E-Da Hospital/I-Shou University, Kaohsiung, Taiwan; ^6^ Department of Pathology, E-Da Hospital/I-Shou University, Kaohsiung, Taiwan; ^7^ Department of Internal Medicine, E-Da Hospital/I-Shou University, Kaohsiung, Taiwan

**Keywords:** disabled-2, survival, recurrence, esophageal cancer

## Abstract

Patients with esophageal squamous cell carcinomas (ESCCs) have poor survival and high recurrence rate, but lack a prognostic biomarker. Disabled-2 (DAB2) is a crucial tumor suppressor, but its roles in ESCCs are uncertain. We investigated whether low DAB2 expression in ESCCs could lead into tumor progression and poor prognosis. Our results found patients with low-DAB2 expression ESCCs had significantly larger tumor size, deeper tumor invasion depth, lymph node metastasis, worse survival, and higher recurrence rate (*P*<0.05). The Cox-regression model revealed low-DAB2 expression was an independent factor of poor survival (*P*<0.05), and also of tumor recurrence with the predictive performance superior to clinical TNM stage (*P*<0.05). Low-DAB2 cancer cells, validated by DAB2 knockdown or over-expression, had higher phosphorylated ERK and migration abilities, which could be suppressed by ERK inhibitor treatment. TGF-β-induced epithelial-to-mesenchymal transition (EMT) only existed in the high-DAB2 cells, and related to worse prognosis of high-DAB2 ESCCs (*P*<0.05). In conclusion, DAB2 can suppress the ERK signaling, but correlate to have TGF-β-induced EMT in ESCCs. DAB2 expression could be a biomarker to identify patients with worse survival and high recurrence. Our data suggest DAB2 expression can stratify patients in need of aggressive surveillance and with possible benefit from anti-ERK or anti-TGF-β therapies.

## INTRODUCTION

Esophageal cancer is a highly lethal disease, causing more than 400,000 deaths per year in the world [1]. In the Asia-Pacific region, esophageal squamous cell carcinoma (ESCC) is the major disease phenotype with an increased incidence in recent years [1, 2]. Despite recent advances in multi-disciplinary treatment, including radical surgical resection, chemotherapy, and radiotherapy, the 5-year survival rates of patients with ESCC remains less than 30% [2–7]. The high recurrence rate (around 40 to 60%) after curative surgery or definitive chemo-radiation therapy is the leading cause of death for such patients [8–12]. However, we continue to lack prognostic biomarkers to identify the risky patients with tumor recurrence and poor survival.

Disabled-2 (DAB2) is a multifunctional adapter protein with tumor suppressor activity, which may act as a potent negative regulator of multiple signaling pathways, including the ERK [13–15], Wnt/β-catenin [16–18], and TGF-β [19, 20] pathways. DAB2 is also shown to participate in TGF-β induced epithelial-to-mesenchymal transition (EMT) [21–24]. A decreased DAB2 expression has been shown in many types of tumors, including head & neck [20], lung [25], ovarian [26], prostate [27], breast [28], and also esophageal cancers [29]. A recent study revealed the DAB2 expression level can predict metastasis and poor prognosis in human squamous cell carcinomas of the head & neck [20]. Nevertheless, the clinical implication of DAB2 in the ESCC, in eager need of a prognosis biomarker, remains uncertain. This study is thus highly original to illustrate low DAB2 expression level can be a prognostic biomarker for the recurrence and survival of patients with ESCC. Moreover, the study disclosed DAB2 can suppress the ERK signaling, but may correlate to have TGF-β-induced epithelial-to-mesenchymal transition (EMT) in ESCC. The study suggests DAB2 expression may not only stratify patients who require aggressive surveillance, but also point out the cases with potential benefit from anti-ERK or anti-TGF-β therapies for ESCC.

## RESULTS

### Demographic features of the ESCC patients with high- and low-DAB2 expressions

**Figure 1:**
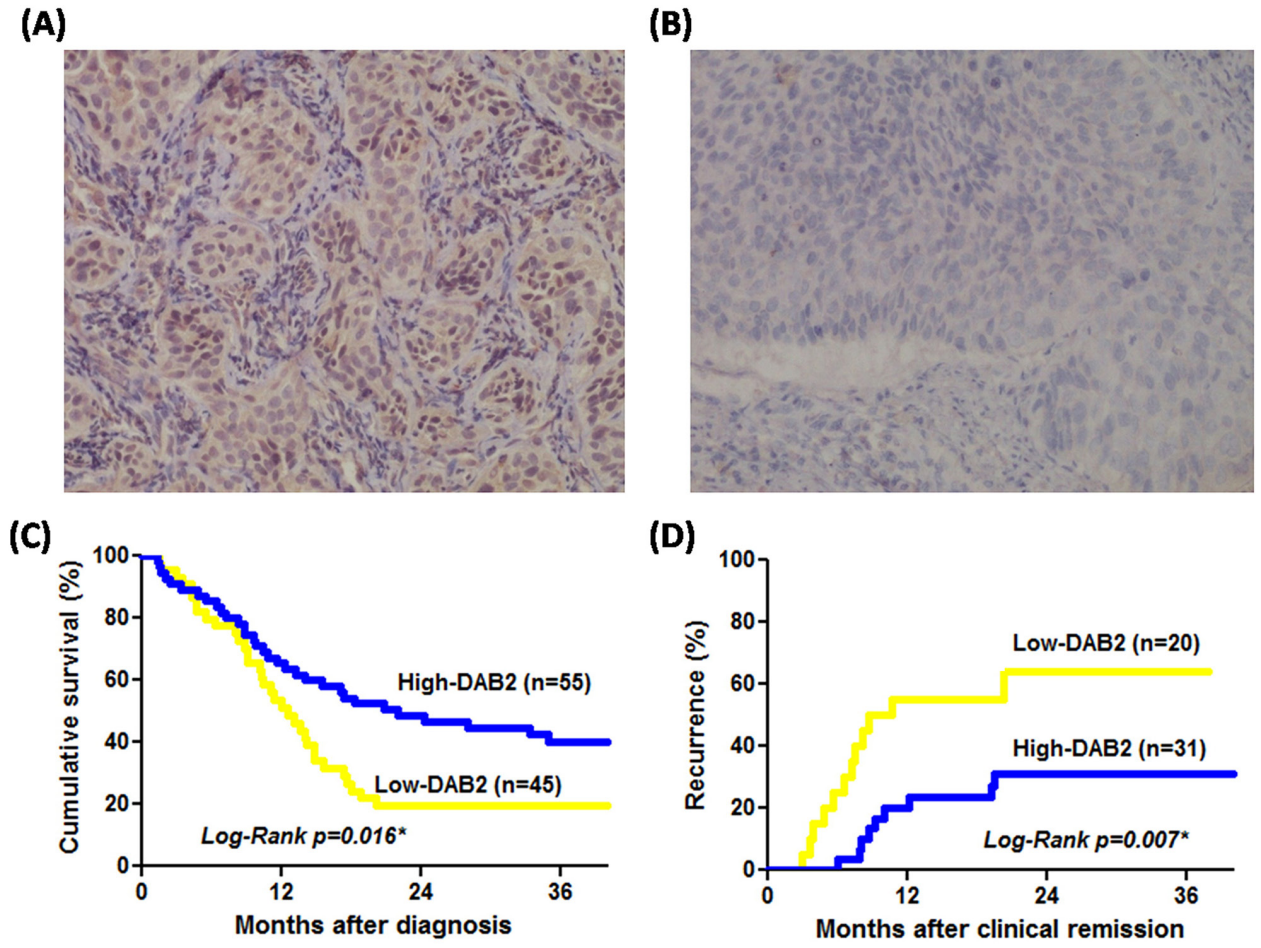
The DAB2 protein of ESCC can be correlated to survival and tumor recurrence rate **A.** High-DAB2 ESCC with cytoplasmic brownish staining of DAB2 protein; **B.** Low-DAB2 ESCC with loss of DAB2 protein (original magnification x 200); **C.** The survival rate by Kaplan-Meier survival curve was lower in patients with low-DAB2 ESCC than in those with high-DAB2 ESCC (*P*=0.016). **D.** The tumor recurrence rate was higher in patients with low-DAB2 ESCC than in those with high-DAB2 ESCC (*P*=0.007).

Forty-five of 100 patients with ESCC defined to have low-DAB2 expression carcinomas (Figure 1A & 1B). In Table 1, as compared to the ESCC with high-DAB2 expression, the ESCC with low-DAB2 expression can be a larger tumor size, more tumor invasion depth, higher frequency of lymph node metastasis, and more advanced clinical stage (*P*<0.05).

**Table 1:** The correlation of DAB2 expression level to the clinical features of ESCCs

Characteristics n (%)		High DAB2	Low DAB2	*p* value
	(n)	(n=55)	(n=45)	
Age, years				
≤ 50	47	28 (60%)	19 (40%)	0.425
> 50	53	27 (51%)	26 (49%)	
Tumor differentiation				
well or moderate	92	49 (48%)	43 (52%)	0.289
poor	8	6 (75%)	2 (25%)	
Primary tumor size				
≤ 4 cm	39	28 (72%)	11 (28%)	**0.008**
> 4 cm	61	27 (44%)	34 (56%)	
Clinical stage				
I or II	23	18 (78%)	5 (22%)	**0.016**
III or IV	77	37 (48%)	40 (52%)	
Invasion depth				
T1-2	24	19 (79%)	5 (21%)	**0.009**
T3-4	76	36 (47%)	40 (53%)	
Lymph node				
negative	15	12 (80%)	3 (20%)	**0.048**
positive	85	43 (51%)	42 (49%)	
Distant metastasis				
negative	72	43 (60%)	29(40%)	0.179
positive	28	12 (43%)	16(57%)	

ESCC: esophageal squamous cell carcinoma

### DAB2 expression and the survival of patients with ESCC

Among these 100 patients, 46 were treated with operation, 26 with definitive chemoradiation therapy and 28 with palliative chemotherapy. The Cox-regression model confirmed the age, tumor size and tumor differentiation were not related with poor survival of ESCC. Both low-DAB2 levels in tumors (HR=1.81, *P*=0.017) and clinical advanced stage (HR=3.49, *P*=0.001) were independent factors of poor ESCC survival (Table 2). In Figure 1C, patients with low-DAB2 ESCC have worse survival than those with high-DAB2 ESCC (*P*=0.016, by log-rank test).

**Table 2:** The factors related to poor survival of patients with ESCC by Cox hazard regression

Factors	Variable	Case No.	95% CI	HR	*p* value
Age	> 50 vs. ≥ 50	53/47	0.81-2.14	1.31	0.321
Tumor size	> 5cm vs. ≥ 5cm	47/53	0.82-2.17	1.34	0.619
Differentiation	Poorly vs. moderate	8/92	0.62-3.34	1.44	0.360
ESCC stage	III, IV vs. I, II	77/23	1.72-7.09	3.49	**0.001**
DAB2	Low vs. High	45/55	1.11-2.96	1.81	**0.017**

Abbreviations: HR, hazard ratio; CI, confidence interval; ESCC: esophageal squamous cell carcinoma.

### DAB2 expression levels and ESCC recurrence

After initial treatment for the 100 ESCC patients, there were 51 patients defined to have achieved clinically complete remission. Among them, 40 received operation-based treatment and 11 definitive chemoradiation therapy with curative intent. There were 20 of 51 (41.2%) patients which developed tumor recurrence at either local or distant sites during the follow-up period. In Table 3, the Cox-regression model revealed that low-DAB2 expression in the pretreatment tumor of patients with ESCC achieved clinical complete remission could be an independent factor to identify patients with recurrence of ESCC (HR: 2.57, *P*=0.041). The predictive performance of low-DAB2 on ESCC tissue is even more superior to the clinical TNM stage or treatment modalities (Table 3). In Figure 1D, the patients with low-DAB2 ESCC had a higher recurrence rate than those with high-DAB2 ESCC (*P=*0.007 by Log-Rank test).

**Table 3:** The independent factors to predict tumor recurrence by Cox hazard regression

Factors	Variable	Case No.	95% CI	HR	*p* value
***Univariate***					
Age	>50 vs. ≧ 50	21/30	0.50-2.85	1.20	0.683
Tumor size	>4cm vs. ≧ 4cm	29/22	0.52-2.88	1.23	0.643
Differentiation	Poor vs. moderate	5/46	0.31-5.74	1.33	0.700
ESCC stage	III vs. I, II	30/21	0.98-7.33	2.68	0.055
Treatment	Definitive CRT vs. OP-based*	11/40	1.01-5.89	**2.44**	**0.047**
DAB2	Low vs. High	20/31	1.31-7.46	**3.12**	**0.010**
***Multivariate***					
ESCC stage	III vs. I, II	30/21	0.57-4.88	1.66	0.357
Treatment	Definitive CRT vs. OP-based*	11/40	0.80-4.92	1.98	0.142
DAB2	Low vs. High	20/31	1.04-6.36	**2.57**	**0.041**

Abbreviations: HR, hazard ratio; CI, confidence interval; ESCC: esophageal squamous cell carcinoma; OP-based*, indicated the patient had received esophagectomy; CRT: concurrent chemoradiation.

### Low disabled-2 expression promotes tumor progression via activation of ERK signaling

**Figure 2:**
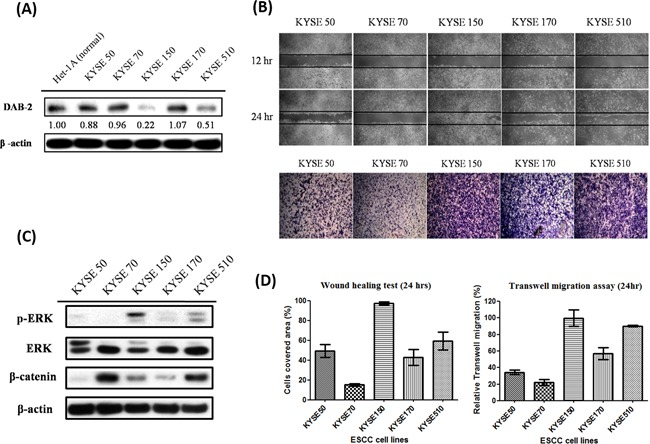
The low-DAB2 esophageal cancer cells had a higher phosphorylated ERK (p-ERK) expression and migration abilities **A.** The western blot analysis of DAB2 protein in five ESCC cell lines (KYSE) and human normal esophageal squamous epithelial cell line (Het-1A). **B.** The wound healing assay (upper panel) and transwell migration assay (lower panel) to evaluate the horizontal and vertical migration abilities in ESCC cell lines. **C.** TheERK, p-ERK and β-catenin levels in ESCC cell lines. **D.** The quantitative representation of migration abilities in ESCC cell lines.

The DAB2 expression levels of five human ESCC cell lines (KYSE-50, KYSE-70, KYSE-150, KYSE-170, KYSE-510) and a human normal esophageal squamous epithelial cell line (Het-1A) were assessed by western blot (Figure 2A). The KYSE-150 and KYSE-510 ESCC cells showed low-DAB2 expression, as compared to normal epithelial cell with high DAB2 expression (Figure 2A). The low-DAB2 expression cell lines (KYSE-150 and KYSE-510) constituted higher migration properties in the cell horizontal and vertical migration abilities by wound closure test and transwell migration assay (Figure 2B).

**Figure 3:**
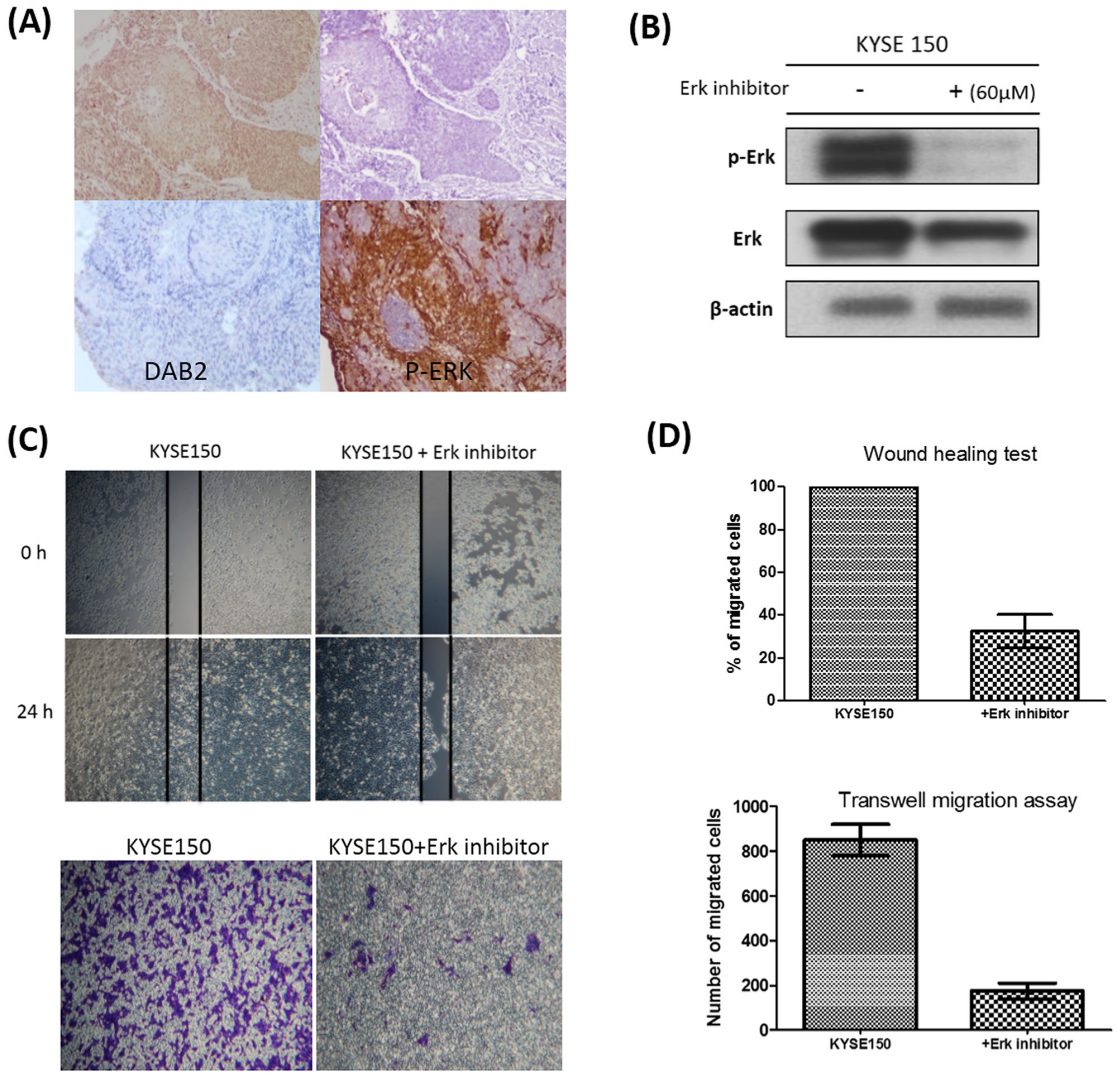
The associations between DAB2 and p-ERK in clinical tumor samples and cell lines **A.** The high-DAB2 cancer (upper panel) had lower p-ERK (upper-right panel) expression, evaluated by immunohistochemistry in clinical tumor samples. The low-DAB2 cancer had higher p-ERK level (lower panel). **B.** The western blot confirmed the suppression of Erk phosphorylation by Erk inhibitor; **C.** The wound healing test (upper panel) and transwell migration assay (lower panel) showed the treatment with ERK inhibitor suppress cancer cell motility of low-DAB2 cell lines; **D.** The quantitative representation of migration abilities in low-DAB2 ESCC cell lines before and after ERK inhibitor treatment.

To explore the underlying mechanisms, we investigated whether the higher invasive property of low-DAB2 cancer was attributed to up-regulation of ERK or Wnt/β-catenin pathways. We found low-DAB2 cancer cells were associated with higher expressions of phosphorylated ERK 1/2 (p-ERK 1/2) (Figure 2C). The DAB2 expressions in cells were inversely correlated with phosphorylated ERK and the ESCC migration abilities (Figure 2D). Similarly, in clinical tumor tissues (Figure 3A), the low-DAB2 ESCC tended to have higher phosphorylated ERK (*P*=0.02). However, the DAB2 expression level did not correlate with β-catenin expression. These findings suggest that low-DAB2 cancers may constitute higher migration abilities via the up-regulation of ERK signaling.

**Figure 4:**
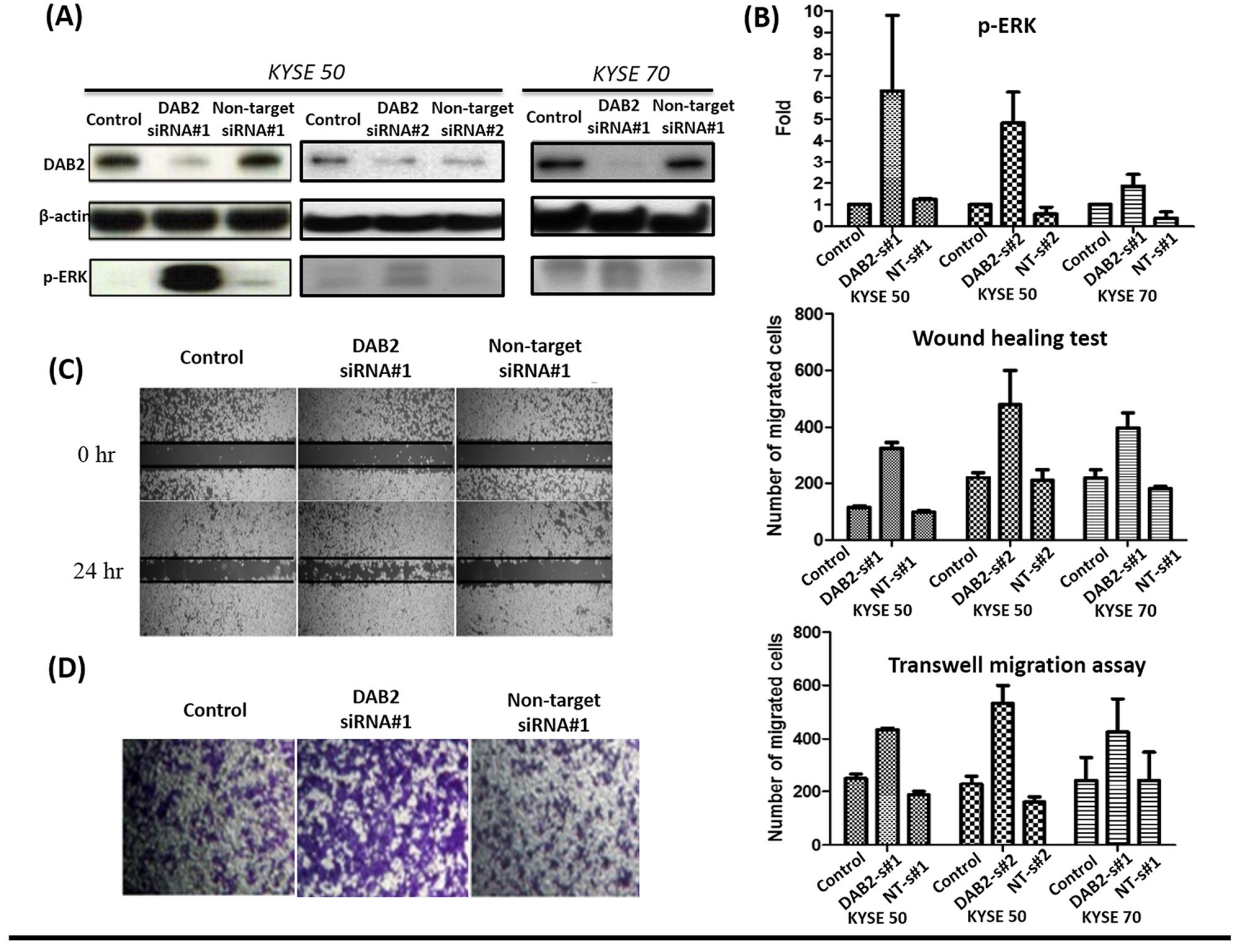
The down-regulation of DAB2 by siRNA in high-DAB2 cell lines promoted ERK phosphorylation and cell motility **A.** Western blot confirmed down-regulation of DAB2 by siRNA in KYSE-50 and KYSE-70 cell lines. **B.** DAB2 down-regulation promoted ERK phosphorylation. The wound healing **C.** and transwell migration assay **D.** showed DAB2 down-regulation promoted cancer cell migration (Abbreviation: s#1: siRNA#1;s#2: siRNA#1; NT, Non-target).

**Figure 5:**
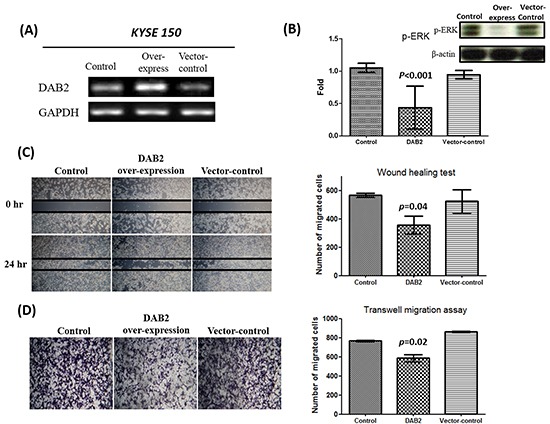
The over-expression of DAB2 can suppress ERK phosphorylation and cancer cell motility **A.** Reverse transcription PCR confirmed the over-expression of DAB2 in Low-DAB2 (KYSE-150) cell line. **B.** DAB2 over-expression inhibited ERK phosphorylation. **C, D.** The wound healing and transwell migration assay showed DAB2 over-expression suppressed cell migration.

We further performed knockdown studies for high-DAB2 cell lines (KYSE-50, KYSE-70) by siRNA targeted against DAB2. In Figure 4, the down-regulation of DAB2 by siRNA targeting increased cancer cell motility and ERK phosphorylation. In Figure 5, the over-expression of DAB2 in the low-DAB2 cell line (KYSE-150) suppressed the ERK phosphorylation and cancer cell motility. Treating with Erk inhibitors (Merk Millipore, FR180204; 60μM) to the low-DAB2 cell line (KYSE-150) can inhibit the cancer cell migration (Figure 3B–3D).

### Low-DAB2 expression level has no EMT

**Figure 6:**
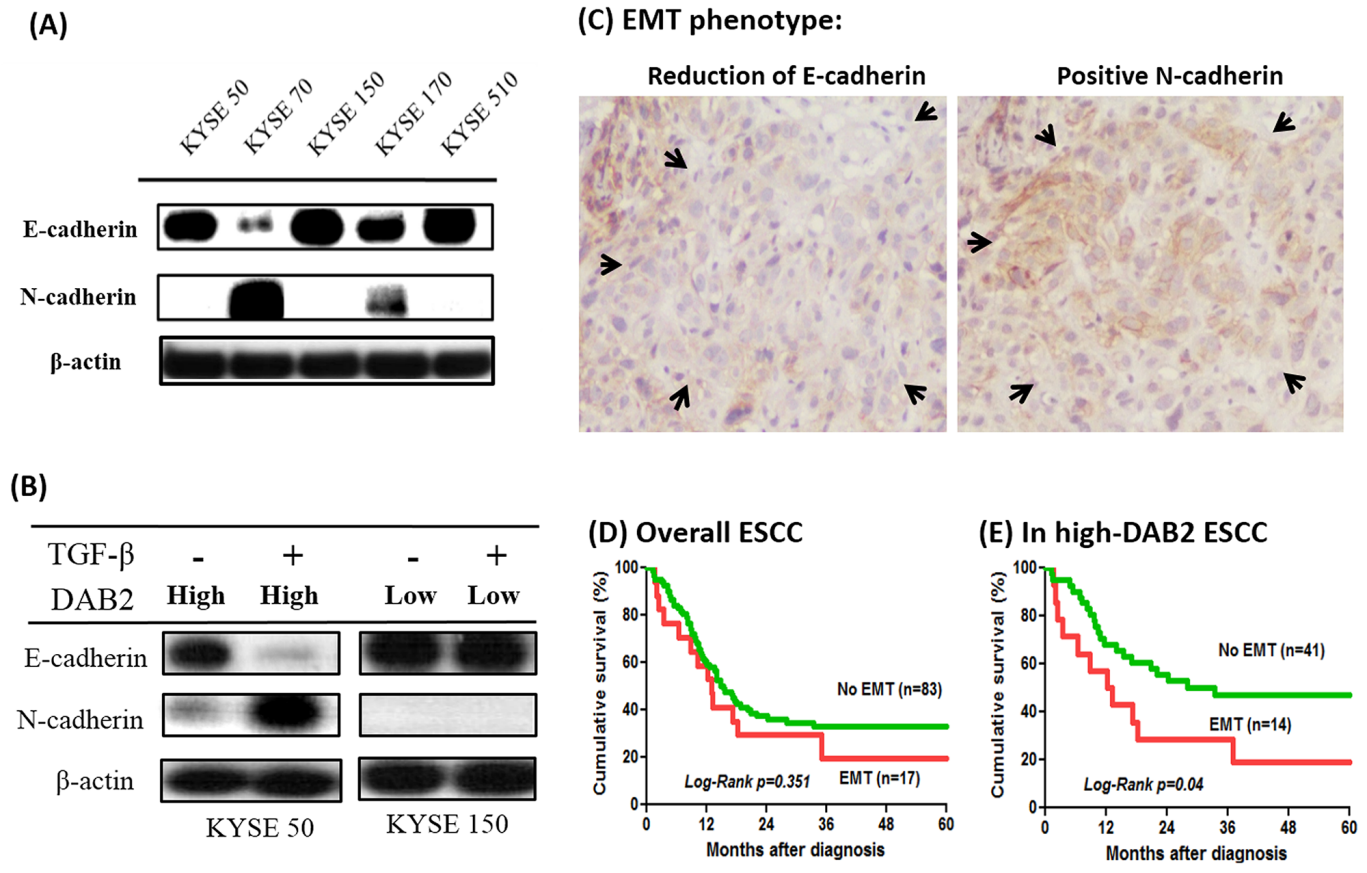
The high-DAB2 esophageal cancer cell had the presence of epithelial-mesenchymal transition (EMT) **A.** Western blot showed EMT phenotype appeared only in high-DAB2 cell lines, KYSE-70 & -170. **B.** Treatment with TGF-β (5 ng/ml) for high-DAB2 (KYSE-50) and low-DAB2 (KYSE-150) ESCC cells, respectively. TGF-β can induce N-cadherin and β-catenin expression in KYSE-50, but not in KYSE-150. **C.** EMT phenotype with reduced E-cadherin and increased N-cadherin expression (arrow) in clinical tumor tissues by immunohistochemistry. The survival analysis by Kaplan-Meier survival curves showed EMT had survival impact on the patients with high-DAB2 cancers (*p*=0.04) **D.** but not in low-DAB2 cancers **E.**

Because DAB2 participates in the TGF-β-induced EMT [21–23], this study tested whether DAB2 expression correlated with the EMT phenotype of ESCC. Our study illustrated EMT phenotype only appeared in the high-DAB2 ESCC cell lines, but not in the low-DAB2 ones (Figure 6A). The data suggested the higher cancer migration ability of low-DAB2 cancer cell lines should be EMT-independent manner.

In Figure 6B, the high-DAB2 (KYSE-50) cells treated with TGF-β can induce EMT phenotype. In contrast, the low-DAB2 (KYSE-150) cells treated with TGF-β cannot induce EMT. Similarly, in primary tumor tissues (Figure 6C), EMT phenotypes were more common in high-DAB2 ESCC than in low-DAB2 ones (25.5% [14/55] vs. 6.7% [3/45], *P*=0.016). Moreover, EMT was significantly correlated with a poor survival for ESCC in high-DAB2 cancers (*p*=0.04), but not in low-DAB2 cancers (Figure 6D & 6E).

### Methylation status of DAB2 promoter in ESCC

**Figure 7:**
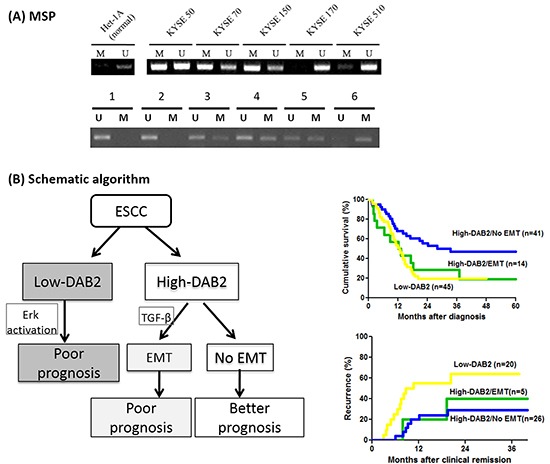
The methylation status of DAB2 promoter and the schematic algorithm demonstrated the three clinical ESCC phenotypes **A.** The analysis of *DAB2* promoter methylation by MSP in the ESCC cell lines (upper panel) and representative primary tumors (lower panel). U and M refer to the un-methylated and the methylated PCR reactions, respectively. **B.** The schematic algorithm demonstrated the three clinical ESCC phenotypes, defined by DAB2 and EMT expressions, and their corresponding survival and tumor recurrence rates.

The study further validate whether aberrant hypermethylation of the promoter in *DAB2* gene may correlate to low DAB-2 expression in ESCC. Because there were 53 CpG dinucleotides in the *DAB2* promoter region, we further evaluated the possibility of methylation-mediated gene silencing of *DAB2* promoter in 45 low-DAB2 ESCC patients by the methylation-specific PCR (Figure 7A). However, only 13 out of 45 (29%) patients with low-DAB2 ESCC had aberrant *DAB2* promoter hypermethylation. Moreover, the methylation status of DAB2 promoter was not well correlated to DAB2 expression in ESCC cell lines (Figure 7A). Some high-DAB2 cell lines (KYSE-50, KYSE-70) even were demonstrated with DAB2 promoter hypermethylation. The data suggests that other mechanisms beyond to *DAB2* promoter hypermethylation can regulate DAB2 expression.

## DISCUSSION

This study provides the first integrated investigation of DAB2 in ESCC at both cellular and clinical levels. We illustrated low-DAB2 level cancer cells constitute an invasive property, probably via activation of ERK signaling. A low-DAB2 expression can be an independent biomarker of worse survival and high recurrence for ESCC, even with better predictive performance than clinical TNM stages.

Decrease of DAB2 protein may occur early since the ESCC development, and sustain to drop-down along with tumorigenic pathway [29]. However, there remains without available data to verify the clinical significance of DAB2 down-regulation in ESCC. In Table 1, the low-DAB2 expression of ESCC tissue was significantly associated with a larger tumor size, a deeper tumor invasion, and a higher frequency of lymph node metastasis. Furthermore, such a low-DAB2 expression tumor may lead into a worse survival. To the best of our knowledge, this is the first study to explore the clinical significance and provided clinical evidence that DAB2 may participate in the process of ESCC progression and may correlate with survival.

DAB2 is a well-established tumor-suppressor and a cytoplasmic adaptor molecule, has been shown to link cell surface receptors to downstream signal transduction, especially the ERK [13–15], Wnt/β-catenin [16–18] and TGF-β signaling pathway [19, 20]. DAB2 can suppress ERK phosphorylation [13]. Our study also demonstrates DAB2 function as a repressor of ERK signaling in ESCC cell lines, *i.e.* low-DAB2 cells can associate to higher levels of ERK phosphorylation, and vice versa. Activation of ERK signaling is a frequent event in tumorigenesis, and has been implicated in cell migration, and angiogenesis, events that are essential for metastasis [33, 34]. In addition, ERK had been reported to phosphorylate HIF-1α, resulting in the induction of VEGF expression, and promoting angiogenesis [33, 35]. Activation of the ERK pathway can also link to expression of MMP-9 [36], which may degrade the extracellular matrix to lead into a worse prognosis of ESCC as shown in our previous study [37]. Taken together, this evidence supports the reason of why low-DAB2 ESCC may have a poor survival and a high risk of recurrence.

DAB2 may serves as a negative regulator of canonical Wnt/β-catenin signaling by stabilizing the β-catenin degradation complex [16, 17]. However, our study did not disclose that DAB2 cannot inhibit the β-catenin accumulation in ESCC cell lines. Our data was highly original to show the EMT phenotype could be only disclosed in high-DAB2 ESCC, but not in low-DAB2 ESCC. This data supported the previous reports to confirm DAB2 shall be involved to TGF-β induced EMT [21–24].

As EMT can link to aggressive, invasive and metastatic behavior of various cancer types [38, 39], including ESCC [40, 41], our study further tested whether EMT phenotype alone can be related with the survival of ESCC. Only in the case of high-DAB2 ESCC, but not in low-DAB2 ESCC, the presence of EMT can determine a worse prognosis of ESCC. Accordingly, DAB2 shall carry on a dual-role manner during tumor progression. The first is that DAB2 serves as suppressor of ERK signaling. Alternatively, it may bridge TGF-β receptors to induce EMT process in a subset of patients. We thus suggested the patients with ESCC could be divided into three phenotypes (Figure 7B): 1> low-DAB2, 2> high-DAB2 with EMT, and 3> high-DAB2 without EMT; with corresponding 2-year overall survival rate as 20%, 30% and 60%, respectively. Such differentiated phenotypes shall be helpful for clinicians to categorize the risk of ESCC with regards to survival.

The silencing of *DAB2* gene expression can be modified by epigenetic changes [20, 42, 43]. The aberrant *DAB2* promoter methylation was detected in 13 of 45 (29%) patients with low-DAB2 ESCC in our study. This data suggested *DAB2* promoter hypermethylation just decreased DAB2 expression in a subset of ESCC. There should be other mechanisms responsible to low DAB2 in ESCC, especially for those with unmethylated *DAB2* promoters.

Based on COX hazard regression analysis (Table 3), low-DAB2 expression of ESCC is even superior to clinical tumor staging to predict the recurrence after treatment. Our data illustrated that DAB-2 expression can be a convenient biomarker to select high-risk ESCC patients to receive more intensive treatment before recurrence occurs. In addition, specific blockade of the ERK pathway has been suggested as an attractive pharmacological target to limit malignant progression and metastasis [33, 44, 45]. Our data illustrated the Erk inhibitors suppressed cell migration in the low-DAB2 cancer cell lines. These data supported ESCC with low-DAB2 expression, commonly associated with high ERK signaling, may be good candidates to receive ERK-targeted therapeutics.

In conclusion, the DAB2 expression levels in ESCC can serve as clinical biomarker to identify patients with poor survival and high-risk of recurrence. ESCC with low-DAB2 expression, or those with high-DAB2 and EMT, shall not only has a worse prognosis, but also provide a guidance to select the suitable target therapy in future.

## MATERIALS AND METHODS

### Patients and study design

This study consecutively enrolled 100 adults with histology-confirmed esophageal squamous cell carcinomas from January 2009 to January 2012. The study protocol conformed to the 1975 Declaration of Helsinki and was approved by the institutional review boards of EDa Hospital (EMRP-20101N) and National Cheng Kung University Medical Center (BR-100-087). Each patient signed their written informed consent to participate in this study, and provide the pre-treatment cancer tissues for the DAB2 expression validation by immunohistochemistry. The clinical staging was verified by computed tomography (CT) scans and endoscopic ultrasound (EUS) to follow the TNM classification system by the American Joint Committee on Cancer [30]. Each study participant was treated after a multi-disciplinary evaluation following the National Comprehensive Cancer Network (NCCN) guidelines. For the patients with stage I or stage II ESCC, curative tumor resection were suggested. For those with stage III cancer, surgery with or without pre-operative chemoradiation therapy were performed. For patients who did not have curative surgery, the initial treatment for the ESCC was definitive concurrent chemoradiation therapy or palliative chemotherapy using platinum-based regimens. We further defined the “clinical complete remission” as the esophageal tumor cannot be detected by endoscopy, histology from endoscopic biopsy, CT, and PET after treatment with curative intent. All of the patients received regular follow-up and the post-treatment survival was evaluated by regular clinic visits, medical records, or telephone contact.

### Immunohistochemistry for DAB2 expression, Erk phosphorylation and EMT phenotype

The esophageal tumor tissue was fixed in 10% buffered formalin, embedded in paraffin, and serially sectioned at 4*μ*m thickness. To stop endogenous peroxidase activity, the specimen was immersed for 20mins in 3% hydrogen peroxide and then pretreated with Dako Cytomation Target Retrieval Solution (Dako, Carpinteria, CA, USA) for antigen retrieval. The tissue section was treated with primary antibody against DAB2 (Catalog sc-13982, Santa Cruz CA, at 1:100 dilution) or against E-cadherin (Cell Signaling, MA, USA, at 1:400 dilution) or against N-cadherin (Abcam, MA, USA, at 1:400 dilution) or phospho-ERK (Cell Signaling, MA, USA, at 1:200 dilution), respectively. Then the sections were incubated overnight in a humidified chamber at 4°C. The Vectastain Elite ABC Kit (Vector Laboratories Inc., Burlingame, CA, USA) was used for blocking, linkage, and labeling for staining according to the manufacturer's instructions. The Dako Cytomation Liquid DAB Substrate Chromogen system was used as chromogen. The section was then counterstained with hematoxylin. The pathologist blinded to patients’ background scored the staining of DAB2, phospho-ERK, E-cadherin, and N-cadherin expressions.

The expression levels of DAB2 protein in the cytoplasm of tumor cells were scored as the intensity of staining and the percentage of positive-stained cells. The surrounding non-neoplastic stroma served as an internal control for each slide. The staining “intensity” measurements were classified into strong “2”, weak “1”, or negative “0” staining. The staining “percentage” measurements in the tumor tissue were graded as “3” if >60% tumor cells were immunostaining-positive; “2” for 30–60%; “1” for 5–30% and “0” if <5% were positive. Then the data of DAB2 expression were defined by “intensity x percentage”, and further dichotomized into either low-DAB2 (“intensity x percentage”≤1) or high-DAB2 (“intensity x percentage” >1) carcinomas, respectively (Figure 1). For those defined as low-DAB2 cancers, a second staining from another tumor section was done.

Semi-quantitative analysis of phospho-ERK staining was assessed as 0, 1+, 2+, and 3+. Grade 0 is defined as the complete absence or weak phospho-ERK immunostaining in <5% of the tumor cells; grade 1+ is focal positivity in 5-25% of the tumor cells; grade 2+ is frequency staining in 25-50% of the tumor cells; and grade 3+ is frequency staining in >50% of the tumor cells. For E-cadherin, cancer cells were divided into two groups: preserved expression, which indicates cells with the same level of expression as that of normal epithelium, and reduced expression, which indicates cells with weak or absent expression compared with normal epithelium. N-cadherin expression was defined as a positive membranous staining using nerve bundle as internal positive control. The epithelial-to-mesenchymal transition (EMT) phenotype was defined as concomitantly reduced expression of E-cadherin to plus a positive expression of N-cadherin.

### Cell culture, plasmids and small interference RNA transfection

The five human esophageal squamous carcinoma cell lines (KYSE-50, KYSE-70, KYSE-150, KYSE-170, KYSE-510) were purchased from the DSMZ-German Collection of Microorganisms and Cell Cultures (Braunschweig, Germany). All cancer cell lines were cultured in RPMI-1640 medium (Gibco, USA), supplemented with 10% fetal bovine serum. A virus transformed human normal esophageal squamous epithelial cell line (Het-1A) [31] was obtained from the American Type Culture Collection (Manassas, VA, USA) and was maintained in bronchial epithelial cell growth medium (BEGM), supplemented by bronchial epithelial growth media kit (Lonza, Walkersville, MD). All cells were incubated at 37°C in 5% CO_2_ humidified atmosphere. GFP-tagged DAB2 (NM_001343) Human cDNA ORF Clone was purchased from Origene Inc (Cat. No. RG200481). Cell lines were co-transfected with pCMV6-AC-GFP-DAB2 plasmids. Small interference RNA#1 (siRNA#1, siGENOME Human DAB2 [1601] siRNA SMART pool) and siRNA#2 (Dharmacon^TM^, pre-designed siRNA; Sense: 5′ C.A.G.C.A.A.A.G.C.A.G.U.U.G.A.G.A.A.U.U.U 3′; Antisense: 5′ A.U.U.C.U.C.A.A.C.U.G.C.U.U.U.G.C.U.G.U.U 3′) targeted against DAB2 were used for knockdown studies. Cell lines were transfected with siRNA using Lipofectamine 2000, as recommended by the manufacturers (Invitrogen). In parallel, cells untreated and transfected with non-targeting siRNA were used as control.

### Western blot analysis

Cells were washed with ice-cold phosphate-buffered saline (PBS) and lysed in ice-cold radio-immunoprecipitation assay lysis buffer (Sigma, St. Louis, MO, USA). The lysates were then fractionated and separated on 10 % sodium dodecyl sulfate–polyacrylamide gel electrophoresis (SDS-PAGE). The proteins on the gels were transferred to polyvinylidene fluoride (PVDF) membrane (Millipore, Billerica, MA, USA). Then the blots were probed with an antibody specific to DAB2 (Santa Cruz Biotechnology, MA, USA), E-cadherin (Cell Signaling, Danvers, MA, USA), N-cadherin (Abcam, Cambridge, MA, USA), β-catenin (Abcam), phospho-ERK (Cell Signaling), ERK (Cell Signaling), and appropriate secondary antibodies. The labeled bands were subsequently detected by enhanced chemiluminescence. For each sample, band intensities were normalized to β-actin.

### Wound closure motility assay and transwell migration assay

Wound closure assay was performed using the IBIDI^TM^ Culture Inserts system. KYSE cell lines (5×10^5^ cells/ml; 70 μl) were seeded into the two wells of the culture insert and incubated at 37°C, 5% CO_2_ for 16 hours. Then the culture inserts were gently removed to create a cell-free gap of ~500 μm. Cells migrating into the gap were evaluated under an inverted microscope and the images were collected and analyzed using ImageJ software (National Institutes of Health, Bethesda, MD, USA). Transwell migration assay was performed using CoStar Tranwell chambers (8 μm pore size, Corning, NY, USA). Cells (1×10^5^/well) were seeded in the upper chambers of the wells in 300 μl serum-free medium, and the lower chambers were filled with 700 μl medium containing 10% fetal bovine serum to induce cell migration. The chamber was incubated at 37°C for 24 hours. At the end of incubation, the cells in the upper surface of the membrane were removed with a cotton swab. Cells migration to the lower surface of the membrane were fixed with methanol and stained with crystal violet. The images were obtained and the cells were counted with a microscope.

### Methylation-specific PCR to define the methylation status of DAB2 promoter

Genomic DNA was prepared from primary tumor samples and cancer cell lines. The methylation status of CpG-island in *DAB2* promoter was analyzed using methylation-specific PCR (MSP) [32]. Genomic DNA (1 μg) was subjected to modification with sodium bisulphite, using the EZ DNA methylation kit (Zymo) according to the manufacturer's instruction. Modified DNA was subjected to MSP utilizing the primers and conditions [20]. The methylation-specific primers were 5′-GACCGAAAACTTCGAAACCGCGCGA-3′ as the forward primer and 5′-GGGGTTT TTTGCGTCGTTGTAGCGC-3′ as the reverse primer, respectively. The unmethylation- specific primers were 5′-ACCAACCAA AAACTTCAAAACCACACAA-3′ as the forward primer and 5′-GTGGGGTTTTTTGTG TTGTTGTAGTGT-3′ as the reverse primer [20].

### Statistical analysis

The statistics were performed with SPSS software (SPSS for Windows, version 18.0; SPSS Inc., Chicago, IL). The clinical characteristics were compared by χ2 test or Student's *t* test between low- and high-DAB2 ESCCs. The study size was at least 100, assuming low and high DAB2 expression distributed in near half. The cumulative survivals of study groups were shown by Kaplan-Meier curves, and their differences were assessed by two-tailed log-rank test. The COX proportional hazard model assess whether DAB2 expression can be independent to predict the survivals and recurrence of ESCC. Correlations between values were evaluated by Spearman's rank correlation. Significance was values of *P* < 0.05.
